# A Prospective Study of Aspirin Use and the Risk of Gastrointestinal Bleeding in Men

**DOI:** 10.1371/journal.pone.0015721

**Published:** 2010-12-29

**Authors:** Edward S. Huang, Lisa L. Strate, Wendy W. Ho, Salina S. Lee, Andrew T. Chan

**Affiliations:** 1 Division of Gastroenterology and Hepatology, Department of Medicine, Massachusetts General Hospital and Harvard Medical School, Boston, Massachusetts, United States of America; 2 Division of Gastroenterology, Department of Medicine, University of Washington, Seattle, Washington, United States of America; 3 Division of Digestive Diseases, Department of Medicine, David Geffen School of Medicine, University of California Los Angeles, Los Angeles, California, United States of America; 4 Department of Medicine, Northwestern University Feinberg School of Medicine, Chicago, Illinois, United States of America; 5 Channing Laboratory, Department of Medicine, Brigham and Women's Hospital and Harvard Medical School, Boston, Massachusetts, United States of America; University of Modena and Reggio Emilia, Italy

## Abstract

**Background and Aims:**

Data regarding the influence of dose and duration of aspirin use on risk of gastrointestinal bleeding are conflicting.

**Methods:**

We conducted a prospective cohort study of 32,989 men enrolled in the Health Professionals Follow-up Study (HPFS) in 1994 who provided biennial aspirin data. We estimated relative risk of major gastrointestinal bleeding requiring hospitalization or a blood transfusion.

**Results:**

During 14 years of follow-up, 707 men reported an episode of major gastrointestinal bleeding over 377,231 person-years. After adjusting for risk factors, regular aspirin use (≥2 times/week) had a multivariate relative risk (RR) of gastrointestinal bleeding of 1.32 (95% confidence interval [CI], 1.12–1.55) compared to non-regular use. The association was particularly evident for upper gastrointestinal bleeding (multivariate RR, 1.49; 95% CI, 1.16–1.92). Compared to men who denied any aspirin use, multivariate RRs of upper gastrointestinal bleeding were 1.05 (95% CI 0.71–1.52) for men who used 0.5–1.5 standard tablets/week, 1.31 (95% CI 0.88–1.95) for 2–5 aspirin/week, 1.63 (95% CI, 1.15–2.32) for 6–14 aspirin/week and 2.40 (95% CI, 1.10–5.22) for >14 aspirin/week (*P_trend_*<0.001). The relative risk also appeared to be dose-dependent among short-term users <5 years; *P_trend_*<.001) and long-term users (≥5 years; *P_trend_* = 0.015). In contrast, after controlling for dose, increasing duration of use did not appear to be associated with risk (*P_trend_* = 0.749).

**Conclusions:**

Regular aspirin use increases the risk of gastrointestinal bleeding, especially from the upper tract. However, risk of bleeding appears to be more strongly related to dose than to duration of use. Risk of bleeding should be minimized by using the lowest effective dose among short-term and long-term aspirin users.

## Introduction

Randomized trials have demonstrated that aspirin lowers the risk of myocardial infarction among patients with prior cardiovascular disease as well as those with cardiovascular risk factors [1], [2], [3]. Despite its therapeutic benefits, the limiting factor for aspirin use has been its association with gastrointestinal toxicity, particularly gastrointestinal bleeding. However, evidence regarding the influence of aspirin dose on the risk of bleeding has been conflicting as noted by a recent consensus statement from the American College of Cardiology Foundation (ACCF) task force [4]. Furthermore, pooled data in meta-analyses also showed contradictory results [5], [6], [7].

Similarly, the effect of duration of aspirin use on risk of gastrointestinal bleeding remains unclear. Some data suggest that over time the gastrointestinal mucosa adapts to the adverse effects of aspirin. This hypothesis has been supported by human studies which have suggested that shorter duration of aspirin use was associated with increased risk of gastrointestinal bleeding [8], [9]. However, other studies show that bleeding risk is cumulative over time [10]. These inconsistent results may be due to heterogeneous study populations, small sample sizes, data on only a limited range of doses, and relatively short duration of follow-up.

Thus, we examined the influence of aspirin use on the risk of gastrointestinal bleeding among men enrolled in the Health Professionals Follow-up study (HPFS) who provided detailed and updated information on aspirin use over 20 year follow-up. This cohort allows for a comprehensive examination of aspirin dose based on prospectively collected data within the context of a range of other lifestyle risk factors.

## Methods

### Study Population

The HPFS is a prospective cohort of 51,529 U.S. male dentists, optometrists, osteopaths, podiatrists, pharmacists and veterinarians, who returned a health questionnaire in 1986. Subsequently, biennial questionnaires were mailed to update information on diet, aspirin use and medical diagnosis with a follow-up rate exceeding 90%. Return of the questionnaire was considered to imply informed consent. The institutional review board at the Harvard School of Public Health approved this study. We also obtained written informed consent to review the medical records.

### Assessment of Aspirin Use

In the 1986 questionnaire and every two years thereafter, we inquired if men used aspirin, two or more time per week (“e.g. Anacin, bufferin, Alka-Seltzer”), and other anti-inflammatory medications (“e.g. Motrin, Indocin, Naprosyn, Dolobid”). Individual non-steroidal anti-inflammatory drug (NSAID) types were not collected. Beginning in 1992, we also asked men the average frequency of intake and also number of aspirin tablets used (in categories). Early in the study, most men used standard-dose aspirin tablets of 325 mg [11]. However, to reflect overall secular trends in consumption of low-dose, or baby aspirin, after 1992, we asked participants to convert intake of four baby aspirin to one adult tablet. Thus, dose category of 0.5 to 1.5 tablets/week was equivalent to 81 milligrams/day (mg/day); 6–14 tablets/week was equivalent to 325 mg/day. Beginning in 2000, questionnaires specifically asked usual doses used by participants (50–99 mg, 100–249 mg, 250–349 mg and ≥350 mg). The reasons for aspirin use were not assessed for the entire cohort, but a supplementary questionnaire was sent in 1993 to a sample of 211 men who reported aspirin use from 1986 to 1990 (88% response). The major reasons for use were cardiovascular disease, 25.4%; to decrease risk for cardiovascular disease, 58.4%; headaches, 25.4%; joint or musculoskeletal pain, 33.0%; and other reasons, 7.0% [11].

### Ascertainment of Outcome

In 2006 and 2008, we asked participants to report any episodes of gastrointestinal bleeding which required hospitalization or blood transfusion, a definition consistent with previous studies [12]. Participants were also asked to provide the anatomic location of the bleed (esophagus, stomach, duodenum, colon/rectum, other, unknown) and the year of the bleed (in categories) over the entire cohort study period. We classified cases into upper and lower gastrointestinal bleeding; upper bleeding was defined as bleeding originating from the esophagus, stomach, and duodenum; lower bleeding was defined as bleeding arising from the colon or rectum. As a validation, we verified accuracy of self-reports by reviewing medical records among a subsample of 239 men who reported bleeding in which we archived blood specimens in 1993–95 for a separate study of genetic risk factors for bleeding. The baseline characteristics of the cohort of men who provided a blood specimen were generally similar to men who did not. Two study physicians (gastroenterologists) blinded to exposure information, reviewed and extracted the data based from hospital records, discharge summaries, endoscopy reports and pathology reports if biopsy specimens were taken. We found the correlation between self-reported date of diagnosis and confirmed date of diagnosis was 0.87 (*P*-value <0.001). The accuracy of the self-reported classification of upper and lower gastrointestinal bleeding was 93.3% (95% confidence interval [CI], 88.8%–96.4%). The etiology of bleeding was validated based on the reports from endoscopy, bleeding scans or angiograms when available. For lower tract cases, diverticular bleeding was presumed if the active diverticular bleeding site was not located and no other sources of lower gastrointestinal bleeding was identified [13], [14], [15]. A third reviewer was used to resolve discrepancies in assigning etiology of bleeding between the two primary physician reviewers.

### Statistical Analysis

At baseline, we included men who returned the 1994 questionnaire on aspirin use. We excluded those men with a prior history of gastrointestinal bleeding, cancer or peptic ulcer disease. We also excluded those cases of bleeding related to cancer or post-polypectomy complications or without a known date of diagnosis. After these exclusions, 32,989 men were eligible for analysis. Person-time for each participant was calculated from the date of return of the 1994 questionnaire to the date of their first gastrointestinal bleed, death from any cause, or January 1, 2008, whichever came first. We recognized that participants may have varied their aspirin use over the follow-up time period. Thus, we used time-varying covariates such that each participant contributed person-time according to the aspirin data they provided on each biennial questionnaire. Consistent with previous analyses of this cohort, we used cumulative average number of standard aspirin tablets used prior to each two-year time period to estimate dosage of aspirin use. We defined men who reported using aspirin two or more times per week were defined as regular users where those who reported less aspirin use were defined as non-regular users [11], [16], [17]. We also grouped men according to previously described number of standard (325 mg) aspirin tablets used per week to estimate dosage of aspirin [17]. We examined the duration of aspirin use by the number of years used according to response to all biennial questionnaires prior to each 2-year follow-up interval. We recalculated the duration of use each time there was a cessation in regular aspirin use after the 1986 questionnaire.

We determined the incidence rates of gastrointestinal bleeding for men in a specific category of aspirin use by dividing the number of incident cases by the number of person-years. We computed relative risk (RR) by dividing the incidence rate of bleeding in one category divided by the incidence rate in the reference category. To minimize any potential biases related to either use or avoidance of aspirin, we censored participants after the self-reported diagnosis of cancer during follow-up; these events were not included as end points. We used Cox proportional hazards modeling to control for multiple variables simultaneously and to compute 95% confidence intervals (CI) [4], [18], [19], [20], [21]. We used time-varying covariates in our model with the most updated information for all covariates prior to each 2-year interval. In additional analyses, we also adjusted for other clinical indications for aspirin use and anticoagulants as time-varying covariates in our multivariate model. The linear trend test across categories was calculated by using the median value of each category and modeling it as a continuous variable [22]. We evaluated interactions by assessing the statistical significance of a cross-product interaction term in the model. We used SAS version 9.1.3 (Cary, NC) for all analyses. All *P* values are two-sided, and a *P* value less than 0.05 was considered significant.

## Results

Among the 32,989 eligible men, we documented 707 gastrointestinal bleeding events over 377,231 person years. At baseline, participants who used aspirin tended to be older, had higher body mass indices, and were more likely to have diabetes, hypertension, hypercholesterolemia, coronary artery disease, and osteoarthritis compared to men who denied no aspirin use. Moreover, men who reported aspirin use consumed more alcohol and were more likely to be previous smokers (**Table 1**).

**Table 1:** Baseline characteristics of the study cohort in 1994.

		Aspirin Tablets (325 mg) per week†				
Characteristics		None(n = 14759)	0.5–1.5(n = 5854)	2–5(n = 5994)	6–14(n = 5819)	>14(n = 563)
Age, mean (SD), y		59.9 (9.3)	60.1 (9.1)	61.8 (9.2)	63.0 (9.1)	61.7 (8.8)
Body mass index, mean (SD), kg/m^2^ ‡		25.8 (3.6)	25.9 (3.4)	26.0 (3.5)	26.3 (3.7)	26.6 (3.9)
Current NSAID use, No. (%)§		1721 (11.7)	669 (11.4)	841 (14.0)	815 (14.0)	105 (18.7)
Physical activity, mean (SD), MET/wk		36.5 (41.2)	36.7 (38.2)	38.2 (39.1)	36.2 (39.8)	33.8 (42.3)
Diabetes mellitus, No. (%)		707 (4.8)	272 (4.7)	296 (4.9)	457 (7.9)	40 (7.1)
Hypertension, No. (%)		3798 (25.7)	1745 (29.8)	2097 (35.0)	2484 (42.7)	230 (40.9)
Hypercholesterolemia, No. (%)		5229 (35.4)	2365 (40.4)	2672 (44.6)	3013 (51.8)	244 (43.3)
Coronary artery disease, No. (%)		406 (2.8)	319 (5.5)	479 (8.0)	1175 (20.2)	52 (9.2)
Osteoarthritis, No. (%)		2538 (17.2)	1054 (18.0)	1253 (20.9)	1408 (24.2)	259 (46.0)
Smoking status						
	Past, No. (%)	5865 (39.7)	2445 (41.8)	2654 (44.3)	2908 (50.0)	292 (51.9)
	Current, No. (%)	787 (5.3)	314 (5.4)	284 (4.7)	304 (5.2)	29 (5.2)
Mean alcohol use, mean (SD), g/day		10.2 (14.5)	10.5 (13.7)	11.7 (14.5)	12.1 (15.6)	13.3 (18.4)

† One standard tablet is 325 mg of aspirin.

‡ Body mass index is weight in kilograms divided by the square of the height in meters.

§ Current NSAID use is defined as regular intake of at least 2 times per week.

The overall absolute incidence in our cohort was 1.87 events per 1,000 person-years, within a similar range of prior studies [23]. The absolute risk of major gastrointestinal bleeding among regular aspirin users (≥2 times/week) was 2.31 events per 1000 person-years compared to 1.38 events among nonregular users. We observed a statistically significant higher risk for bleeding among regular aspirin users compared with non-regular users even after adjusting for other potential risk factors including body mass index, physical activity, smoking, alcohol intake and concomitant NSAID use (multivariate RR, 1.32; 95% CI, 1.12–1.55) (**Table 2**). The effect appeared to be primarily related to upper gastrointestinal bleeding (multivariate RR, 1.49; 95% CI, 1.16–1.92). For the lower gastrointestinal tract, we found a non-significant association between regular aspirin use and risk of bleeding (multivariate RR, 1.22; 95% CI 0.95–1.56).

**Table 2:** Relative risk of gastrointestinal bleeding according to regular use of aspirin†.

	Non-Regular users	Regular users	*P* Value
**All Cases** *			
Person-years	176496	200735	
No of cases	244	463	
Age-adjusted RR (95% CI)	1.0	1.36 (1.16–1.60)	<.001
Multivariate RR (95% CI)‡	1.0	1.32 (1.12–1.55)	0.001
**Upper GI Bleeding**			
Person-years	176496	200735	
No of cases	99	204	
Age-adjusted RR (95% CI)	1.0	1.53 (1.19–1.97)	<.001
Multivariate RR (95% CI)‡	1.0	1.49 (1.16–1.92)	0.002
**Lower GI Bleeding**			
Person-years	176496	200735	
No of cases	110	193	
Age-adjusted RR (95% CI)	1.0	1.26 (0.99–1.62)	0.063
Multivariate RR (95% CI)‡	1.0	1.22 (0.95–1.56)	0.124

† Regular aspirin use is defined as consumption of ≥2 times per week**.** Non-regular use is defined as consumption of <2 times per week. Relative risks (RR) are compared to non-regular users as reference group.

*Includes 101 individuals with unknown or unspecified location of GI bleeding.

‡ Multivariate RR model is adjusted for age, NSAID use (yes or no), smoking status (never, past, current), body mass index (<21. 21–22.9, 23–24.9, 25–29.9, ≥30 kg/m^2^), exercise (<1.7, 1.7–4.5, 4.6–10.5, 10.6–22.0, ≥22.1 mets/week), alcohol (0, 0.1–4.9, 5–14.9, ≥15 g/day).

Among the 239 cases for whom we reviewed complete medical records, we found that the 53.1% of cases were bleeds which originated from the upper gastrointestinal tract, 38.5% originated from lower gastrointestinal tract, and 8.4% did not have a clearly localized site of bleeding. The most common causes of upper gastrointestinal bleeding were ulcers (64.0%), inflammation (gastritis or duodenitis) (20%), Mallory-Weiss tear (4.0%), arteriovenous malformations (2.4%), Dieulafoy's lesions (1.6%), and portal hypertension (1.6%). In the lower gastrointestinal tract, the most common causes of bleeding were diverticular (66.7%), inflammation (colitis) (18.0%), arteriovenous malformations (3.9%), and ulcer (3.9%). An etiology was not identified in 4.8% and 3.9% of bleeding arising from the upper tract and lower tract, respectively.

We observed an increasing risk of gastrointestinal bleeding with more frequent aspirin use even after adjustment for other bleeding risk factors (*P_trend_*<.001) (**Table 3**). Compared to nonusers, men who used aspirin most frequently (≥6 days/week) had a multivariate RR of 1.39 (95% CI, 1.14–1.69) for all gastrointestinal bleeding and 1.56 (95% CI, 1.16–2.11) for upper gastrointestinal bleeding. Although we also observed a progressively greater risk of lower gastrointestinal bleeding among men who used aspirin more frequently (*P_trend_* = 0.021), this apparent effect was attenuated after adjusting for other bleeding risk factors (*P_trend_* = 0.055).

**Table 3:** Relative risk of gastrointestinal bleeding according to frequency of aspirin use†.

	Days per week of use					
	None	<2	2–3	4–5	≥6	*P* trend**
**All Cases** *						
Person-years	117552	58944	32742	24607	143386	
No of cases	163	81	42	52	369	
Age-adjusted RR (95% CI)	1.0	1.02 (0.78–1.34)	0.91 (0.64–1.28)	1.51 (1.09–2.07)	1.46 (1.20–1.77)	<.001
Multivariate RR (95% CI)‡	1.0	1.02 (0.78–1.34)	0.90 (0.64–1.28)	1.51 (1.09–2.07)	1.39 (1.14–1.69)	<.001
**Upper GI Bleeding**						
Person-years	117552	58944	32742	24607	143386	
No of cases	66	33	22	21	161	
Age-adjusted RR (95% CI)	1.0	1.02 (0.67–1.56)	1.17 (0.72–1.91)	1.55 (0.94–2.56)	1.63 (1.21–2.21)	<.001
Multivariate RR (95% CI)‡	1.0	1.01 (0.66–1.54)	1.17 (0.72–1.91)	1.54 (0.93–2.55)	1.56 (1.16–2.11)	<.001
**Lower GI Bleeding**						
Person-years	117552	58944	32742	24607	143386	
No of cases	74	36	18	22	153	
Age-adjusted RR (95% CI)	1.0	1.01 (0.67–1.51)	0.87 (0.52–1.46)	1.34 (0.83–2.18)	1.34 (1.00–1.79)	0.021
Multivariate RR (95% CI)‡	1.0	0.99 (0.66–1.48)	0.85 (0.51–1.44)	1.33 (0.82–2.17)	1.27 (0.94–1.70)	0.055

† Relative risks (RR) are compared to those without any aspirin use as reference group.

*Includes 101 individuals with unknown or unspecified location of GI bleeding

**P trend calculated using median aspirin frequency of each category as continuous variable

‡ Multivariate RR model is adjusted for age, NSAID use (yes or no), smoking status (never, past, current), body mass index (<21. 21–22.9, 23–24.9, 25–29.9, ≥30 kg/m^2^), exercise (<1.7, 1.7–4.5, 4.6–10.5, 10.6–22.0, ≥22.1 mets/week), alcohol (0, 0.1–4.9, 5–14.9, ≥15 g/day).

Multivariate RR model is adjusted for aforementioned variables as well as aspirin dose (continuous use in tablets per week).

Since men who used aspirin more frequently were more likely to have used the drug for longer periods, we examined the influence of duration of aspirin use with risk (**Table 4**). Compared to men who denied any aspirin use, participants who reported greater than 10 years of aspirin use had an age-adjusted RR of 1.26 (95% CI, 0.99–1.62; *P_trend_* = 0.050) for gastrointestinal bleeding. However, after adjusting for dose of aspirin as well as other potential confounders, the effect was no longer significant (*P_trend_* = 0.749). For upper gastrointestinal bleeding, we found a similar relationship between duration of use and risk of bleeding (*P_trend_* = 0.012). However, after we controlled for the aspirin dose, we again found that the association was no longer evident (*P_trend_* = 0.641). No significant association was seen between duration of use and lower gastrointestinal bleeding.

**Table 4:** Relative risk of gastrointestinal bleeding according to duration of regular aspirin use†.

	Duration of Continuous Use (years)				
	None	1–5	6–10	>10	*P* trend**
**All Cases** *					
Person-years	176496	103931	56853	39950	
No of cases	244	241	130	92	
Age-adjusted RR (95% CI)	1.0	1.40 (1.16–1.68)	1.38 (1.10–1.72)	1.26 (0.99–1.62)	0.050
Multivariate RR (95% CI)‡	1.0	1.35 (1.12–1.62)	1.36 (1.09–1.70)	1.18 (0.92–1.52)	0.131
Multivariate RR + Dose (95% CI)§	1.0	1.19 (0.95–1.48)	1.18 (0.91–1.53)	1.01 (0.75–1.35)	0.749
**Upper GI Bleeding**					
Person-years	176496	103931	56853	39950	
No of cases	99	101	61	42	
Age-adjusted RR (95% CI)	1.0	1.47 (1.10–1.96)	1.68 (1.20–2.34)	1.50 (1.03–2.18)	0.012
Multivariate RR (95% CI)‡	1.0	1.44 (1.07–1.91)	1.67 (1.19–2.33)	1.41 (0.97–2.06)	0.027
Multivariate RR + Dose (95% CI)§	1.0	1.18 (0.84–1.66)	1.33 (0.90–1.96)	1.11 (0.71–1.71)	0.641
**Lower GI Bleeding**					
Person-years	176496	103931	56853	39950	
No of cases	110	102	49	42	
Age-adjusted RR (95% CI)	1.0	1.33 (1.00–1.75)	1.15 (0.81–1.62)	1.27 (0.88–1.83)	0.345
Multivariate RR (95% CI)‡	1.0	1.27 (0.96–1.69)	1.12 (0.79–1.59)	1.19 (0.82–1.72)	0.522
Multivariate RR + Dose (95% CI)§	1.0	1.20 (0.86–1.68)	1.05 (0.70–1.57)	1.10 (0.71–1.70)	0.932

† Relative risks (RR) are compared to those without any continuous aspirin use as reference group.

*Includes 101 individuals with unknown or unspecified location of GI bleeding.

**P trend calculated using median aspirin dose of each category as continuous variable.

‡ Multivariate RR model is adjusted for age, NSAID use (yes or no), smoking status (never, past, current), body mass index (<21. 21–22.9, 23–24.9, 25–29.9, ≥30 kg/m^2^), exercise (<1.7, 1.7–4.5, 4.6–10.5, 10.6–22.0, ≥22.1 mets/week), alcohol (0, 0.1–4.9, 5–14.9, ≥15 g/day).

§ Multivariate RR model is adjusted for aforementioned variables as well as aspirin dose (continuous use in tablets per week).

We found that gastrointestinal bleeding risk progressively increased with higher doses (*P_trend_*<.001) (**Table 5**). In particular, men who used dose equivalent to 325 mg/day (6–14 tablets/week) had higher risk of upper gastrointestinal bleeding (multivariate RR, 1.63; 95% CI, 1.15–2.32) than men who used dose equivalent to 81 mg/day (0.5 to 1.5 tablets/week) (multivariate RR 1.05; 95% CI, 0.71–1.54). We also observed that the dose-response effect remained significant for all gastrointestinal bleeding (*P_trend_*<.001) and upper gastrointestinal bleeding (*P_trend_*<.001) even after controlling for duration of use. For lower tract bleeding, we did not find a significant relationship between aspirin dose and risk of bleeding. Specifically, we found that dose category equivalent to 81 mg/day had a similar risk in lower gastrointestinal bleeding (multivariate RR, 1.24; 95% CI, 0.87–1.76) compared to dose category equivalent to 325 mg/day (multivariate RR, 1.31; 95% CI, 0.92–1.86). We also performed analysis beginning from 2000 when specific doses were first asked (**Table 6**). Individuals who took 50–99 mg or baby aspirin equivalent had a multivariate RR of 1.21 (95% CI, 0.94–1.57); 250–349 mg or standard aspirin equivalent had a multivariate RR of 1.69 (95% CI, 1.26–2.27). These results did not differ appreciably when restricted to only daily users (multivariate RR, 1.17; 95% CI, 0.89–1.53 for 81 mg equivalent and multivariate RR, 1.67; 95% CI, 1.20–2.33 for 325 mg equivalent).

**Table 5:** Relative risk of gastrointestinal bleeding according to dose of aspirin use†.

	Aspirin Tablets (325 mg) per week					
	None	0.5–1.5	2–5	6–14	>14	*P* trend**
**All Cases** *						
Person-years	117552	76515	65668	111768	5728	
No of cases	163	137	91	301	15	
Age-adjusted RR (95% CI)	1.0	1.21 (0.96–1.53)	1.00 (0.77–1.30)	1.49 (1.22–1.82)	1.77 (1.03–3.03)	<.001
Multivariate RR (95% CI)‡	1.0	1.20 (0.95–1.51)	0.99 (0.76–1.29)	1.42 (1.16–1.74)	1.70 (0.99–2.90)	<.001
Multivariate RR + Duration (95% CI)§	1.0	1.22 (0.96–1.54)	1.02 (0.78–1.34)	1.49 (1.18–1.87)	1.80 (1.03–3.13)	<.001
**Upper GI Bleeding**						
Person-years	117552	76515	65668	111768	5728	
No of cases	66	48	47	134	8	
Age-adjusted RR (95% CI)	1.0	1.07 (0.73–1.56)	1.30 (0.89–1.90)	1.68 (1.24–2.29)	2.41 (1.14–5.09)	<.001
Multivariate RR (95% CI)‡	1.0	1.04 (0.71–1.52)	1.30 (0.89–1.91)	1.61 (1.18–2.20)	2.36 (1.11–5.00)	<.001
Multivariate RR + Duration (95% CI)§	1.0	1.05 (0.71–1.54)	1.31 (0.88–1.95)	1.63 (1.15–2.32)	2.40 (1.10–5.22)	<.001
**Lower GI Bleeding**						
Person-years	117552	76515	65668	111768	5728	
No of cases	74	64	35	125	5	
Age-adjusted RR (95% CI)	1.0	1.26 (0.89–1.77)	0.83 (0.55–1.25)	1.38 (1.02–1.87)	1.27 (0.51–3.17)	0.050
Multivariate RR (95% CI)‡	1.0	1.24 (0.88–1.74)	0.81 (0.54–1.23)	1.30 (0.96–1.77)	1.18 (0.47–2.94)	0.128
Multivariate RR + Duration (95% CI)§	1.0	1.24 (0.87–1.76)	0.82 (0.53–1.25)	1.31 (0.92–1.86)	1.19 (0.46–3.04)	0.164

† Relative risks (RR) are compared to non-users as reference group.

*Includes 101 individuals with unknown or unspecified location of GI bleeding.

**P trend calculated using median aspirin dose of each category as continuous variable.

‡ Multivariate RR model is adjusted for age, NSAID use (yes or no), smoking status (never, past, current), body mass index (<21. 21–22.9, 23–24.9, 25–29.9, ≥30 kg/m^2^), exercise (<1.7, 1.7–4.5, 4.6–10.5, 10.6–22.0, ≥22.1 mets/week), alcohol (0, 0.1–4.9, 5–14.9, ≥15 g/day).

§Multivariate RR model is also adjusted for aspirin duration (continuous use in years).

§§Multivariate RR model is also adjusted for aspirin frequency (median aspirin frequency of each category as continuous variable).

**Table 6:** Aspirin dose and risk of gastrointestinal bleeding (2000–08).

	Aspirin Dose		
	None	81 mg†	325 mg§
**All Cases** *			
Person-years	71094	41190	21256
No of cases	139	115	71
Age-adjusted	1.0	1.24 (0.96–1.60)	1.73 (1.29–2.32)
Multivariate-adjusted‡	1.0	1.21 (0.94–1.57)	1.69 (1.26–2.27)
**Daily Users**			
Person-years	71094	34663	14988
No of cases	139	94	52
Age-adjusted	1.0	1.18 (0.90–1.54)	1.69 (1.22–2.35)
Multivariate-adjusted‡	1.0	1.17 (0.89–1.53)	1.67 (1.20–2.33)

† Individuals reported taking 50–99 mg of aspirin.

§ Individuals reported taking 250–349 mg of aspirin.

*Includes 49 bleeding cases which were unspecified. Includes non-daily and daily users.

‡ Multivariate RR model is adjusted for age, NSAID use (yes or no), smoking status (never, past, current), body mass index (<21. 21–22.9, 23–24.9, 25–29.9, ≥30), exercise (<1.7, 1.7–4.5, 4.6–10.5, 10.6–22.0, ≥22.1 mets/week), alcohol (0, 0.1–4.9, 5–14.9, ≥15 g/day).

We also examined the effect of dose among intermittent users (1–5 days/week) and daily users (≥6 days/week) of aspirin (**Table 7**). In both groups, the influence of aspirin on gastrointestinal bleeding remained dose-dependent (*P_trend_* = 0.005 among intermittent users and *P_trend_* = 0.007 among daily users). We also found a strong dose-response relationship among both short-term users (<5 years use) (*P_trend_*<.001) and long-term users (≥5 years) (*P_trend_* = 0.015). To assess if duration or frequency of aspirin use significantly modified the association between aspirin dose and bleeding risk, we examined cross-product interaction terms. We did not observe any statistically significant interaction between aspirin dose and either frequency (*P_interaction_* = 0.255) or duration (*P_interaction_* = 0.202).

**Table 7:** Relative risk of gastrointestinal bleeding according to dose of aspirin use†.

	Aspirin Tablets (325 mg) per week					
	None	0.5–1.5	2–5	6–14	>14	*P* trend**
**Intermittent Users (1**–**5 days/week)**						
Person-years	117552	53183	47938	14254	918	
No of cases	163	72	61	39	3	
Age-adjusted RR (95% CI)	1.0	1.03 (0.77–1.36)	0.95 (0.70–1.28)	1.77 (1.23–2.54)	2.08 (0.63–6.86)	0.003
Multivariate RR (95% CI)‡	1.0	1.02 (0.77–1.36)	0.96 (0.71–1.30)	1.67 (1.16–2.42)	2.16 (0.66–7.06)	0.005
**Daily Users (≥6 days/week)**						
Person-years	117552	23332	17729	97514	4811	
No of cases	163	65	30	262	12	
Age-adjusted RR (95% CI)	1.0	1.57 (1.16–2.12)	1.17 (0.78–1.74)	1.47 (1.19–1.82)	1.67 (0.92–3.03)	0.002
Multivariate RR (95% CI)‡	1.0	1.53 (1.13–2.07)	1.13 (0.76–1.69)	1.42 (1.15–1.76)	1.58 (0.87–2.87)	0.007
**Short-Term Users (<5 years)** §						
Person-years	117552	63007	44449	53167	2250	
No of cases	163	104	57	154	7	
Age-adjusted RR (95% CI)	1.0	1.17 (0.91–1.50)	0.95 (0.70–1.30)	1.59 (1.26–2.01)	2.33 (1.08–5.06)	<.001
Multivariate RR (95% CI)‡	1.0	1.15 (0.89–1.48)	0.95 (0.70–1.29)	1.52 (1.20–1.93)	2.16 (0.99–4.70)	<.001
**Long-Term Users (≥5 years)** §						
Person-years	117552	13508	21219	58601	3479	
No of cases	163	33	34	147	8	
Age-adjusted RR (95% CI)	1.0	1.45 (0.98–2.14)	1.12 (0.77–1.64)	1.43 (1.13–1.82)	1.47 (0.71–3.04)	0.008
Multivariate RR (95% CI)‡	1.0	1.42 (0.96–2.09)	1.14 (0.78–1.66)	1.39 (1.09–1.78)	1.49 (0.72–3.09)	0.015

† Relative risks (RR) are compared to non-users as reference group.

‡ Multivariate RR model is adjusted for age, NSAID use (yes or no), smoking status (never, past, current), body mass index (<21. 21–22.9, 23–24.9, 25–29.9, ≥30 kg/m^2^), exercise (<1.7, 1.7–4.5, 4.6–10.5, 10.6–22.0, ≥22.1 mets/week), alcohol (0, 0.1–4.9, 5–14.9, ≥15 g/day).

**P trend calculated using median aspirin dose of each category as continuous variable.

§ Reference group for both short-term and long-term analyses are individuals who reported no use of aspirin (0 years and 0 tablets/week).

In 1994, the baseline prevalence of cardiovascular disease was low with 7.4% of the men having a history of coronary heart disease, 0.8% prior stroke and 0.1% prior atrial fibrillation. In addition, 1.7% had a history of venous thromboembolism (deep vein thrombosis or pulmonary embolism). Through 2008, 13.0% of men had a history of coronary heart disease (of whom 63.8% reported having had a coronary angiography), 1.8% stroke, 0.6% atrial fibrillation, and 3.4% venous thromboembolism. We considered the possibility that these conditions may influence the risk of bleeding according to aspirin use. However, additionally adjusting for the diagnoses of coronary heart disease, atrial fibrillation, stroke and venous thromboembolism did not materially alter our risk estimates (multivariate RR, 1.41; 95% CI, 1.15–1.72). Furthermore, we also considered the influence of warfarin use on gastrointestinal bleeding risk in our cohort. Data on use of warfarin was routinely collected beginning in 1996. In 1996, 2.6% of participants were using warfarin regularly; while in 2004, 5.1% of participants used warfarin. After restricting the analysis to follow-up after 1996, additional adjustment for use of warfarin did not materially alter the risk of bleeding among regular aspirin users (multivariate RR, 1.44; 95% CI, 1.16–1.80).

Similarly, we considered the possibility that concurrent use of proton pump inhibitors or histamine-2 antagonists may influence our results. After additional adjustment for use of proton pump inhibitors and histamine-2 antagonists, the risk of bleeding remained significant (multivariate RR, 1.38; 95% CI, 1.13–1.69).

**Figure 1:**
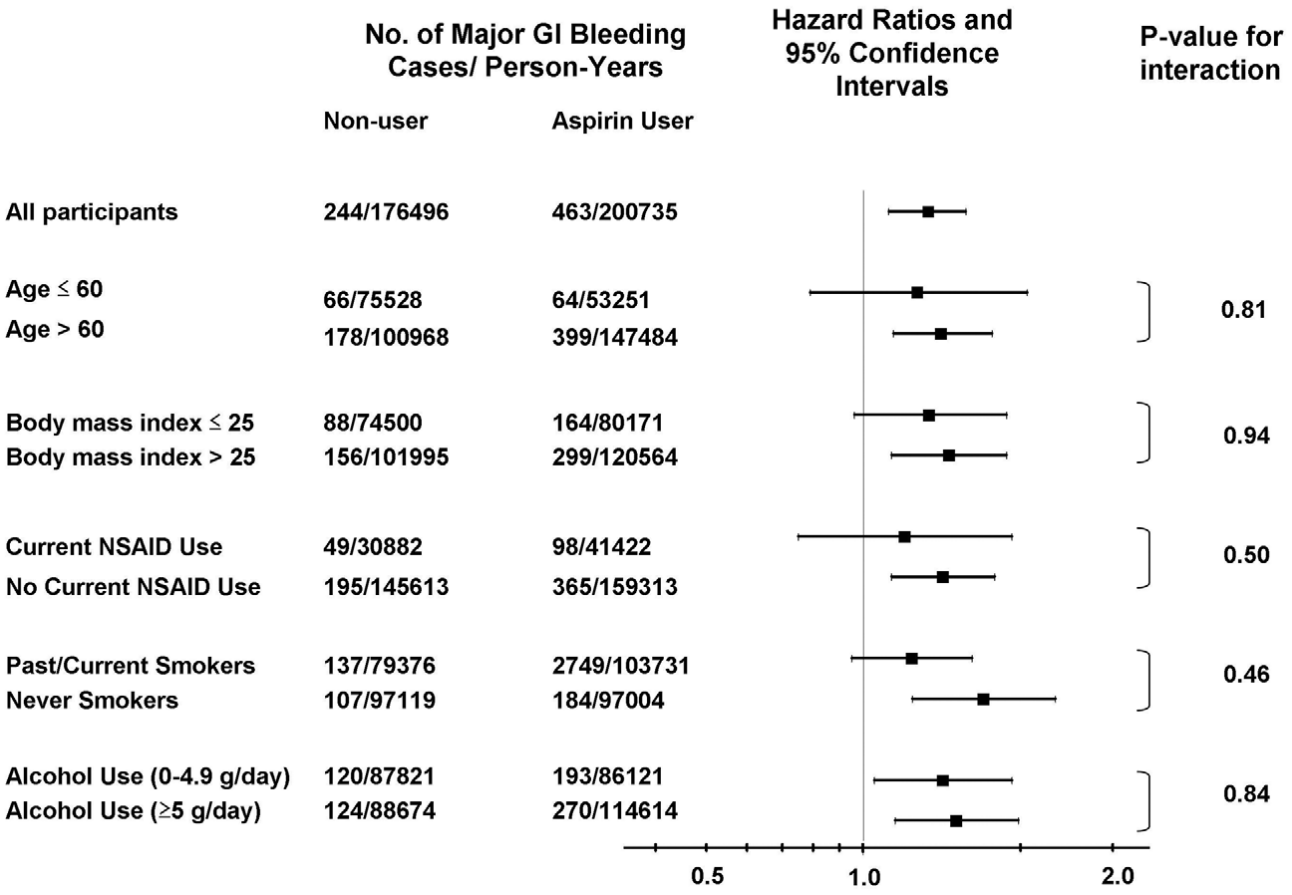
Multivariate-adjusted stratified analyses of gastrointestinal bleeding risk according to aspirin use. Multivariate hazard ratios are adjusted for age (years), NSAID use (yes or no), smoking status (never, past, current), body mass index (<21, 21–22.9, 23–24.9, 25–29.9, ≥30 kg/m^2^), physical activity (<1.7, 1.7–4.5, 4.6–10.5, 10.6–22.0, ≥22.1 mets/week), alcohol (0, 0.1–4.9, 5–14, ≥15 g/day). For each stratified analysis, the stratification variable was omitted from the model.

Finally, we evaluated possible differences in the influence of aspirin according to strata of clinical characteristics. There were no significant differences in the effect of regular aspirin use in the strata defined by age, body mass index, NSAID use, smoking or alcohol use (**Figure 1**).

## Discussion

In this prospective, population-based cohort of men, we found that long-term, regular aspirin use (≥2 times per week) was associated with an increased risk of gastrointestinal bleeding. Moreover, the risk of gastrointestinal bleeding increased with higher doses of aspirin with the greatest risk observed among men who used >14 standard (325-mg) tablets per week. This effect was primarily evident for bleeding originating from the upper gastrointestinal tract. Notably, we observed this dose-response relationship among intermittent users (1–5 days/week) and daily users (≥6 days/week), and among short-term (<5 years) and long-term (≥5 years) users. In contrast, the duration of aspirin use did not appear to be significantly associated with gastrointestinal bleeding after controlling for dose. Furthermore, dose category equivalent to 325 mg/day (6–14 tablets/week) was associated with a higher risk of upper gastrointestinal bleeding (multivariate RR, 1.63; 95% CI 1.15–2.32) compared to dose category equivalent to 81 mg/day (0.5 to 1.5 tablets/week) (multivariate RR, 1.05; 95% CI, 0.71–1.54).

Data from previous controlled trials, case-controlled studies, and meta-analyses generally support our findings that aspirin use is associated with increased risk of major gastrointestinal bleeding [1], [2], [3], [5], [6], [7], [12], [24], [25], [26], [27], [28], [29], [30]. However, previous results from most controlled trials were based on individuals with cardiovascular disease, limiting their generalizability. In addition, case-control studies may overestimate risk due to a retrospective assessment of aspirin use or the selection of a non-representative control population. Many previous studies also have relatively short follow-up time, examine only a few aspirin doses, or have limited data on a range of potential confounding factors.

Several lines of evidence support the dose-dependent effect of aspirin we observed. Animal models show that size of aspirin-mediated gastric lesions are dose-dependent [31]. In addition, higher doses of aspirin more strongly suppress cytoprotective mucosal prostaglandin synthesis [32], [33]. A recent meta-analysis of clinical trials showed that rate of major bleeding events increased with higher doses of aspirin. Patients taking >200 mg aspirin/day had 2.69% chance of having a major gastrointestinal bleeding event compared to 0.97% in patients taking <100 mg/day (*P* = 0.001) [5]. Another study showed that daily doses of 75 mg, 150 mg and 300 mg of aspirin were associated with odd ratios of 2.3 (95% CI, 1.2–4.4), 3.2 (95% CI 1.7–6.5) and 3.9 (95% CI 2.5 to 6.3), respectively [30].

With over 20 years of follow-up, our present study was uniquely able to examine the influence of duration of aspirin use on bleeding risk. Although increasing duration of use was associated with greater risk of bleeding, this effect was no longer evident after accounting for the higher dose typically used by long-term users. These findings are supported by experimental data that have demonstrated that gastric mucosa may adapt to the ulcerogenic properties of aspirin after prolonged exposure [34], [35], [36]. In human studies, several clinical trials have also suggested that aspirin use is associated primarily with gastrointestinal complications early in treatment, supporting the potential for gastrointestinal mucosal adaptation [26], [30], [37], [38], [39].

We did evaluate the effects of other medications known to potentially influence bleeding risk, such as proton pump inhibitors and histamine-2 antagonists. Several studies have shown that proton pump inhibitors can reduce ulcer formation, upper gastrointestinal bleeding and need for surgery [40], [41], [42], [43], [44], [45]. Similarly, endoscopy studies have suggested that histamine-2 antagonists prevent duodenal ulcers and reduce risk of upper gastrointestinal bleeding [42], [46], [47]. In our study, concurrent use of these medications did not appear to significantly influence the risk of bleeding among regular aspirin users. However, these findings should be interpreted with caution given the relatively small proportion of the cohort used these medications over follow-up compared to aspirin users. For example, in 2004, 14.0% and 5.6% were regular users of proton pump inhibitors and histamine-2 antagonists, respectively. Moreover, although proton pump inhibitors and histamine-2 antagonists may reduce risk of aspirin-related bleeding among individuals with preexisting ulcer disease, it remains unclear if these drugs specifically influence risk of initial bleeding or if chronic use of these medications can influence long-term risk [48]. Further studies should examine the potential differential effect of proton pump inhibitors and histamine-2 antagonists on bleeding risk among chronic aspirin users.

Although previous studies have demonstrated an association between aspirin and risk of gastrointestinal bleeding [5], [6], [7], our study differs in several important ways. First, we collected detailed, updated information on aspirin use at a wider range of doses and over longer follow-up than would be feasible to examine in most other studies. Second, we prospectively collected aspirin data prior to diagnosis. Thus, any recall bias would be minimized and likely have attenuated true associations. Third, we were able to estimate several distinct parameters of aspirin use such as dose, duration and frequency of use, making our findings less prone to internal confounding due to correlations among these variables (eg use at higher doses may reflect more consistent use). Fourth, since our participants were all health professionals, the accuracy of self-reported aspirin use is likely to be high and reflect actual consumption. Finally, we used time-varying, biennially updated data on aspirin use and other risk factors in our analysis which minimizes any residual confounding related to changes in risk factors over time.

Our study also has limitations that deserve comment. First, aspirin use was self-selected which may affect the reliability of our results. However, our findings are consistent with widely accepted biological mechanisms in experimental models and the results of prior clinical trials [5], [6], [7]. Furthermore, adjustment for a wide range of potential factors had minimal influence on our findings, suggesting little potential for residual or uncontrolled confounding. Second, assessment of gastrointestinal bleeding did not begin until 2006 which may have led to underascertainment of cases. However, with a high response rate (>90%) and low overall incidence rate of gastrointestinal bleeding, the number of missed cases are likely to be low. Third, since we did not confirm the exact date and cause of each case of gastrointestinal bleeding, misclassification bias is possible. However, among the subset of men for whom we retrieved medical records, we found the self-reported date of diagnosis and location of bleed to be highly accurate. According to our validation subsample, the vast majority of upper gastrointestinal bleeding cases were due to ulcer disease or gastritis/duodenitis, while causes of lower gastrointestinal bleeding were attributed to diverticular disease or colitis. These findings are consistent with other studies of major bleeding [38], [49]. Fourth, our study population consisted of male health professionals, which may limit the generalizability to other population. However, there is little biological reason to expect that differences in the association of aspirin with bleeding would differ by occupation. Moreover, we conducted a similar analysis using the Nurses Health Study, a prospective cohort of women and found consistent results [50]. Finally, we did not specifically inquire about the use of clopidogrel through 2006. However, clopidogrel was not widely used in the U.S. population over the time period of the study (prior to 2008). In our 2008 biennial questionnaire, only 3.7% of individuals reported using this medication. Thus, it is unlikely that concomitant use of clopidogrel over the time period of follow-up could account for our findings.

Although the results of our study are not as definitive as a randomized controlled trial, we believe that such a study would not likely be ethical or feasible since the low incidence of events would require a large number of participants, particularly if multiple aspirin doses are examined. Moreover, since aspirin use for disease prevention requires chronic intake over many years, the prolonged follow-up would be prohibitive.

In conclusion, our findings showed that regular aspirin use is associated with higher risk of gastrointestinal bleeding, especially from the upper tract. Specifically, we found doses equivalent to 325 mg/day was associated with a higher risk of upper gastrointestinal bleeding compared to doses equivalent to 81 mg/day. Moreover, our study suggests that this effect is more strongly related to dose than to duration or frequency of use. These data suggests that minimization of bleeding risk should focus on using the lowest effective dose for both short-term and long-term users.
